# Anti-rheumatic agent auranofin induced apoptosis in chronic myeloid leukemia cells resistant to imatinib through both Bcr/Abl-dependent and -independent mechanisms

**DOI:** 10.18632/oncotarget.2361

**Published:** 2014-08-22

**Authors:** Xin Chen, Xianping Shi, Chong Zhao, Xiaofen Li, Xiaoying Lan, Shouting Liu, Hongbiao Huang, Ningning Liu, Siyan Liao, Dan Zang, Wenbin Song, Quentin Liu, Bing Z. Carter, Q. Ping Dou, Xuejun Wang, Jinbao Liu

**Affiliations:** ^1^ State Key Lab of Respiratory Disease, Protein Modification and Degradation Lab, Departments of Pathophysiology and Biochemistry, Guangzhou Medical University, Guangdong 510182, China; ^2^ Guangzhou Research Institute of Cardiovascular Disease, the Second Affiliated Hospital, Guangzhou Medical University, Guangzhou, Guangdong 510260, People's Republic of China; ^3^ Sun Yat-sen University Cancer Center, State Key Laboratory of Oncology in South China, Collaborative Innovation Center of Cancer Medicine, Guangzhou 510060, China; ^4^ Section of Molecular Hematology and Therapy, Department of leukemia, The University of Texas M.D. Anderson Cancer Center, Houston, TX 77030, USA; ^5^ The Molecular Therapeutics Program, Barbara Ann Karmanos Cancer Institute, and Departments of Oncology, Pharmacology and Pathology, School of Medicine, Wayne State University, Detroit, Michigan 48201-2013, USA; ^6^ Division of Basic Biomedical Sciences, Sanford School of Medicine of the University of South Dakota, Vermillion, South Dakota 57069, USA

**Keywords:** Auranofin, proteasome, chronic myelogenous leukemia, imatinib resistance, Bcr-Abl

## Abstract

Resistance to Imatinib mesylate (IM) is an emerging problem for patients with chronic myelogenous leukemia (CML). T315I mutation in the Bcr-Abl is the predominant mechanism of the acquired resistance to IM and second generation tyrosine kinase inhibitors (TKI). Therefore it is urgent to search for new measures to overcome TKI-resistance. Auranofin (AF), clinically used to treat rheumatic arthritis, was recently approved by US Food and Drug Administration for Phase II clinical trial to treat cancer. In contrast to the reports that AF induces apoptosis by increasing intracellular reactive oxygen species (ROS) levels via inhibiting thioredoxin reductase, our recent study revealed that AF-induced apoptosis depends on inhibition of proteasomal deubiquitinases (UCHL5 and USP14). Here we report that (i) AF induces apoptosis in both Bcr-Abl wild-type cells and Bcr-Abl-T315I mutation cells and inhibits the growth of IM-resistant Bcr-Abl-T315I xenografts *in vivo*; (ii) AF inhibits Bcr-Abl through both downregulation of Bcr-Abl gene expression and Bcr-Abl cleavage mediated by proteasome inhibition-induced caspase activation; (iii) proteasome inhibition but not ROS is required for AF-induced caspase activation and apoptosis. These findings support that AF overcomes IM resistance through both Bcr/Abl-dependent and -independent mechanisms, providing great clinical significance for cancer treatment.

## INTRODUCTION

Chronic myelogenous leukemia (CML) is a myeloproliferative disorder characterized by a reciprocal translocation between chromosomes 9 and 22, resulting in the expression of a fusion oncoprotein, Bcr-Abl, which is found in nearly 95% CML patients [1]. The aberrant tyrosine kinase activity of this chimeric protein is responsible for malignant transformation by activating multiple signal transduction pathways, including the MAPK/ERK cascade, PI3K/Akt, and STATs [2-4]. Activation of these pathways in Bcr-Abl expressing cells results in increased expression of several antiapoptotic proteins, such as Bcl-2, Bcl-xL, Mcl-1, XIAP, thus leading to advantaged cell survival [5-7]. Bcr-Abl tyrosine kinase has been considered as an important target for CML therapeutics [8, 9]. Imatinib mesylate (IM) is the first selective tyrosine kinase inhibitor for cancer therapy approved by the United States Food and Drug Administration (FDA). Clinical studies show that IM is highly effective in newly diagnosed patients with chronic phase CML, and to a less extent, in patents with accelerated and blastic-phase disease [10]. Unfortunately, resistance to IM develops over time and is becoming an emerging problem for CML treatment [11]. Among several mechanisms proposed to explain the IM resistance, amplification and mutation of Bcr-Abl are believed to be the main cause. Approximately 50 point mutations in the Bcr-Abl gene have been identified and associated with clinical resistance to IM. The T315I missense mutation of Bcr-Abl accounting for about 20% of all the point mutations is the most stubborn point mutation impacting on the binding of IM with Bcr-Abl kinase domain and thereby causing IM resistance [12, 13]. To overcome the acquired resistance to IM, new tyrosine kinase inhibitors (TKI) such as nilotinib, dasatinib and INNO-406 have been developed and are effective against most mutations but not the T315I mutation [14-16]. Thus, novel strategies to overcome IM resistance are desperately needed. To this end, ponatinib has been established as a third generation TKI, which suggests that inhibiting Bcr-Abl expression is a promising approach [17].

Auranofin (AF) is a metal phosphine complex that has been used for the clinical treatment of rheumatoid arthritis, following the pioneering studies conducted with gold (I) thiolate compounds [18]. Previous studies have suggested that AF acted as an inhibitor of thioredoxin reductase, which could cause the oxidative damage and modifications of cellular redox status, resulting in over production of reactive oxygen species (ROS), endoplasmic reticulum (ER) stress and apoptosis [19, 20]. AF can exert a strong cytotoxic effect on several different types of neoplastic cells both *in vitro* and *in vivo* [21-23]. The cytotoxic activity of AF along with its relative safe profile in patients warrants the application potential of AF in cancer therapy and other diseases [24, 25]. AF is currently in phase II clinical trials for the treatment of leukemia such as chronic lymphocytic leukemia (http://clinicaltrials.gov/ct2/show/NCT01419691). Most of the previous reports believe that AF induce apoptosis by inhibiting thioredoxin reductase activity and increasing intracellular ROS levels; however, our recent study unravels that AF-induced apoptosis depends on AF-mediated inhibition of proteasomal deubiquitinases (DUBs,UCHL5 and USP14) but not ROS generation [26]. We and others have reported that proteasome inhibition could overcome IM-resistance in CML cells [27, 28], but whether the inhibition of DUBs, especially proteasome-associated DUBs, can overcome IM-resistance has not been reported.

Here, we investigated the antineoplastic effects of AF in both Bcr-Abl wild-type and Bcr-Abl-T315I mutant cell lines and in mouse IM-resistant xenograft models. The results clearly show that AF can efficiently overcome IM-resistance through both Bcr/Abl-dependent and -independent mechanisms that are independent of ROS.

## RESULTS

### AF induces cytotoxicity in both Bcr-Abl wild-type and Bcr-Abl-T315I cells

**Figure 1:**
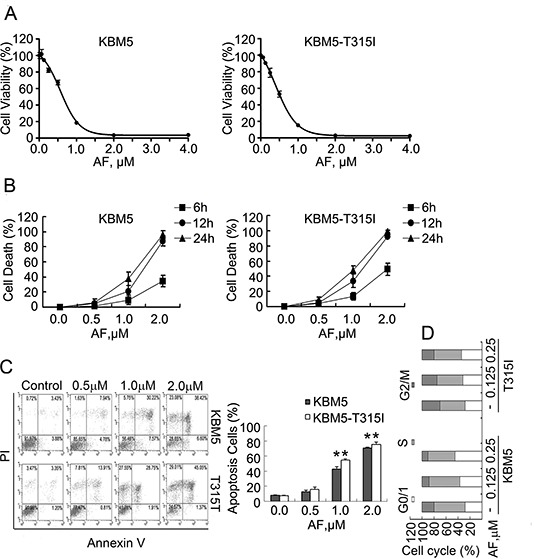
AF induces proliferation inhibition and apoptosis of CML cells **(A)** Dose-response curves of CML cells to auranofin (AF). KBM5 and KBM5-T315I cells were cultured with escalating concentrations of AF for 48 hours. Cell viability was then examined by the MTS assay. Graphs represent data from three independent experiments. Mean±SD (n=3). **(B)** AF treatment induced cell death in CML cells. KBM5 and KBM5-T315I cells were grown in 24-well plates and treated with AF in various concentrations for indicated time periods (6, 12 and 24 hours). Cell death was detected by trypan blue exclusion test. Mean±SD (n=3). **(C)** Induction of apoptosis in CML cells by AF. KBM5 and KBM5-T315I (T315I) cells were treated with the indicated concentrations of AF for 24 hours, the percentage of cells undergoing apoptosis was determined with Annexin V/PI staining by flow cytometry (left). The proportion of cell apoptosis by flow cytometry was summarized (right). Mean±SD (n=3), *P<0.05, *versus* vehicle control. **(D)** Cell cycle arrest in CML cells by AF. KBM5 and KBM5-T315I (T315I) cells were treated with AF for 24 hours and the distribution of the CML cells in different phases of the cell cycle was determination by flow cytometry.

KBM5 (Bcr-Abl wild-type) cells are sensitive to IM while KBM5-T315I (Bcr-Abl-T315I) cells are very resistant to IM [13, 28]. To investigate the effect of AF on the growth of CML cells, KBM5 and KBM5-T315I cells were treated with AF *in vitro* for 48 hours and cell viability was detected by the MTS assay. As shown in Figure 1A, AF dose-dependently decreased the cell viability in KBM5 and KBM5-T315I cell cultures with IC_50_ values of 0.57 and 0.50 μM, respectively.

We next analyzed the dynamics of AF induction of cell death in Bcr-Abl wild-type and T315I mutant cell lines. KBM5 and KBM5-T315I cells were exposed to AF followed by the trypan blue exclusion test, a time- and dose-dependent increasing proportion of cell death was observed by recording the number of trypan blue-positive cells (Figure 1B). Similarly, exposure of KBM5 and KBM5-T315I cells to escalating concentrations of AF resulted in significantly increased Annexin V/PI-positive cells as detected by flow cytometry analysis (Figure 1C), supporting that AF induces apoptosis in CML cells. It was further found that AF induced cell cycle arrest at the G0/G1 phase in both KBM5 and KBM5-T315I cells (Figure 1D).

### AF induces caspase activation in CML cells

**Figure 2:**
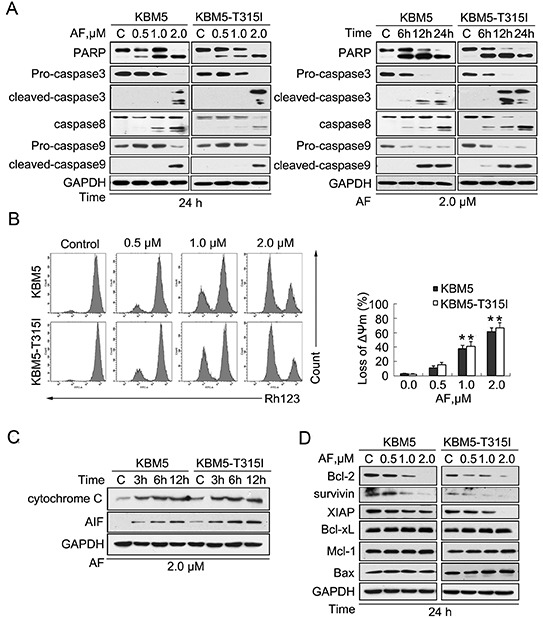
AF induces caspase activation in CML cells **(A)** AF induced PARP cleavage and caspase activation in KBM5 and KBM5-T315I cells. Cells were treated with AF at the indicated concentrations for the indicated durations before harvested and processed for western blot analyses for PARP and caspase-3, -8, -9 cleavage. GAPDH was probed as a loading control. C: vehicle control. **(B)** AF induced down-regulation of mitochondrial membrane potential in KBM5 and KBM5-T315I cells. Cells were treated with 0.5,1.0 and 2.0 μM AF for 24 hours, mitochondrial membrane potential were detected by rhodamine-123 staining with flow cytometry, Mean±SD (n=3), *P<0.05, *versus* vehicle control. **(C)** AF induced cytochrome C and AIF (apoptosis inducing factor) release. KBM5 and KBM5-T315I cells were exposed to 2.0 μM AF for 3, 6 and 12 hours, then cell cytoplasm were extracted by digitonin buffer and the released cytochrome C and AIF were detected with western blot analyses. **(D)** AF decreased the expression of anti-apoptotic proteins in KBM5 and KBM5-T315I cells. Cells were dose-dependently treated with AF for 24 hours. Apoptosis-associated proteins Bcl-2, survivin, XIAP, Bcl-xL, Mcl-1 and Bax were analyzed by Western blot.

KBM5 and KBM5-T315I cells were exposed to AF, followed by measurement of specific apoptosis-associated changes. Western blot analysis showed that AF induced the cleavage of PARP in both dose- and time-dependent manner in these two CML cell lines. Also, the precursor forms of caspase-3, -8 and -9 were decreased while the active forms of caspase-3, -8 and -9 were detected after AF treatment, in parallel to PARP cleavage. These results indicate that AF triggers caspase-dependent CML cell apoptosis (Figure 2A).

It is well known that mitochondria are central to the regulation of apoptosis. Release of cytochrome C and AIF (apoptosis induce factor) from mitochondria to cytoplasm is an indicator of the early stage of apoptosis. As displayed in Figure 2B, the integrity of mitochondrial membranes was decreased in both KBM5 and KBM5-T315I cells after AF treatment, and the release of cytochrome C and AIF to the cytoplasm were elevated in a time-dependent manner in both cell lines (Figure 2C).

To further investigate the mechanism by which AF induces apoptosis, the effect of AF on the expression of other apoptosis-related proteins was examined. As shown in Figure 2D, AF induced a remarkable decline in the expression of anti-apoptotic proteins, including Bcl-2, survivin, and XIAP in both KBM5 and KBM5-T315I cell lines, with less significant changes in the expression of Bcl-xL, Mcl-1 and Bax.

### AF down-regulates Bcr-Abl protein and inhibits its downstream signaling

**Figure 3:**
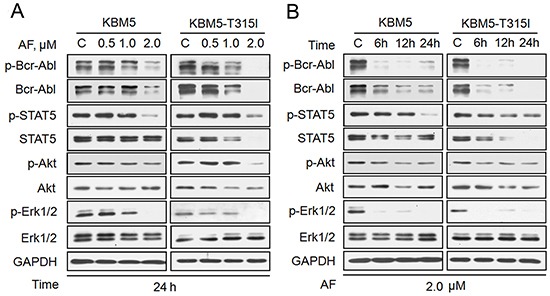
AF induces down-regulation of Bcr-Abl and its downstream signaling proteins KBM5 and KBM5-T315I cells were dose- and time-dependently treated with AF. Bcr-Abl and its downstream proteins were detected by western blot. The dose-dependent changes are shown in A and the time course of the changes is shown in B. GAPDH was used as a loading control. C: control.

We also found that AF down-regulated the levels of total and phosphorylated Bcr-Abl proteins in KBM5 and KBM5-T315I cell lines in both dose- and time-dependent manners (Figure 3A and B). Furthermore, the expression of Bcr-Abl downstream target proteins was also affected by AF. The phosphorylation of STAT5, ERK1/2 and Akt was all significantly decreased, with less dramatic changes in the levels of total ERK1/2 proteins, even though total Akt and STAT5 proteins were decreased. The decreases in total Akt and STAT5 occurred relatively later than the changes of their phosphorylation forms. Taken together, our data demonstrate that AF induces down-regulation of Bcr-Abl protein and inhibits the downstream signaling of Bcr-Abl.

### Bcr-Abl down-regulation by AF is associated with diminished gene expression and caspase-dependent cleavage

**Figure 4:**
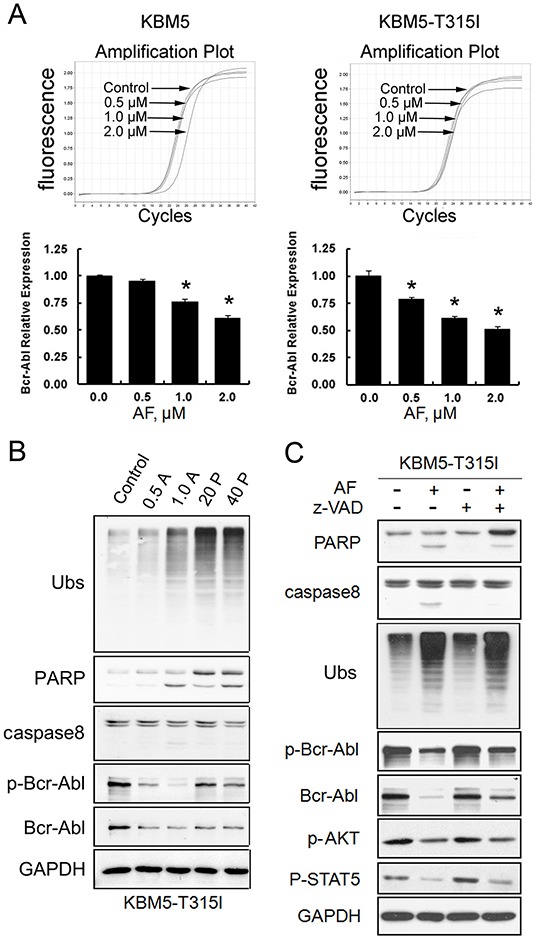
AF-induced Bcr-Abl downregulation is associated with diminished mRNA expression and with caspase activation **(A)** AF decreased mRNA expression of Bcr-Abl. KBM5 and KBM5-T315I cells were exposed to 0.5, 1.0, 2.0 μM AF for 6 hours. The Bcr-Abl mRNA expression was measured by RT-qPCR and its expression level relative to the control was calculated. Mean±SD (n=3), *P<0.05, *versus* control. **(B)** Proteasome inhibition induced Bcr-Abl downregulation. KBM5-T315I cells were treated with either AF (0.5, 1.0 μM) and PS-341(20, 40 nM) for 24 h, then ubiquitinated proteins (Ubs), PARP, caspases-8, Bcr-Abl and p-Bcr-Abl were detected by western blot analyses. GAPDH was used as a loading control. **(C)** AF decreased Bcr-Abl and the downstream signaling proteins in a caspase-dependent manner. KBM5-T315I cells were treated with 2.0 μM AF with/without caspase inhibitor z-VAD-fmk (20 μM) for 6 hours. The total and phosphorylated Bcr-Abl and its downstream proteins were detected by Western blot.

To investigate the mechanism of the AF-mediated Bcr-Abl protein down-regulation in CML cells, we determined the expression of Bcr-Abl at the transcription level. KBM5 and KBM5-T315I cells were treated with increasing concentrations of AF for 6 hours and the steady state Bcr-Abl mRNA levels were examined using RT-qPCR. We found that AF treatment reduced Bcr-Abl mRNA level to the some extent in both cell lines (Figure 4A). The degree of Bcr-Abl mRNA reduction is less dramatic than that of Bcr-Abl protein reduction, suggesting that AF-induced suppression of Bcr-Abl transcription may not account entirely for, but is likely partially responsible for the decreased Bcr-Abl protein levels. This proposition is further supported by comparing the effects of AF and bortezomib on Bcr-Abl expression. As shown in Figure 4B, both DUB inhibitor AF and proteasome inhibitor bortezomib caused the accumulation of ubiquitinated proteins (Ubs), caspase activation, and Bcr-Abl down-regulation but AF seemed to be more potent than bortezomib in downregulating Bcr-Abl and p-Bcr-Abl proteins, consistent with the notion that AF inhibits Bcr-Abl gene expression.

We and others have reported that Bcr-Abl can be cleaved by caspase activation [28, 29]. We observed that pan-caspase inhibitor z-VAD-fmk could inhibit AF-mediated cell death, Bcr-Abl decreases and its downstream event proteins to a certain extent but did not attenuate ubiquitinated protein accumulation (Figure 4C). These results suggest that AF-induced caspase activation is required for downregulation of Bcr-Abl and its downstream events.

### Proteasome inhibition but not ROS induction is required for AF-induced Bcr-Abl downregulation, caspase activation and apoptosis in CML cells

**Figure 5:**
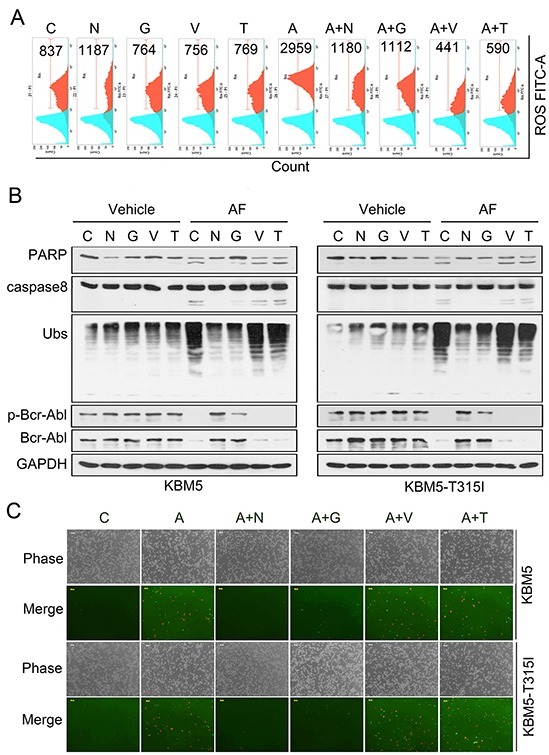
Proteasome inhibition but not ROS is required for AF-mediated Bcr-Abl downregulation, caspase activation and apoptosis **(A)** Both thiol-containing antioxidants (NAC, GEE) and non-thiol-containing antioxidants (Vitamin C, Tbhq) reversed AF-induced ROS generation. KBM5-T315I cells were exposed to AF in the absence or presence of antioxidants for 6 h, ROS was detected with DCF-DA staining by flow cytometry. Typical results were shown. C: control; N: NAC (5 mM); G: GEE (2 mM); A: auranofin (2.0 μM); V: Vitamin C (100 μM); T: Tbhq (10 μM). **(B)** Thiol-containing antioxidants but not non-thiol-containing antioxidants reversed AF-induced proteasome inhibition, caspase activation and Bcr-Abl downregulation in CML cells. KBM5 and KBM5-T315I were pre-treated with antioxidants as in A for half an hour followed by 2.0 μM AF treatment for 6 hours. Cell lysates were analyzed by Western blot analysis using antibodies against ubiquitinated proteins, caspase-8, PARP and Bcr-Abl. GAPDH was used as a loading control. **(C)** Thiol-containing antioxidants but not non-thiol-containing antioxidants reversed AF-induced apoptosis. KBM5 and KBM5-T315I cells were treated as in B, then cell apoptosis was detected with Annexin V/PI staining followed by recording the fluorescent images under an inverted fluorescence microscope. Representive images were shown.

Similar to other cancer cell lines we previously reported [26], we found that AF induced accumulation of ubiquitinated proteins and proteasome-specific substrate proteins in a dose- and time-dependent manner in KBM5 and KBM5-T315I cell lines; AF did not alter the proteasome peptidases in KBM5 and KBM5-T315I cells either (data not shown). It has been believed that AF induces apoptosis *via* ROS generation, which was, however, challenged by our most recent findings [30, 31]. Next we determined the roles of proteasome inhibition and ROS in AF-induced cytotoxicity in CML cells. As shown in Figure 5A, AF indeed induced ROS induction in CML cells. Both thiol-containing antioxidants (NAC, GEE) and non-thiol-containing antioxidants (Tbhq, Vitamin C) could inhibit AF-induced ROS generation (Figure 5A); however, only thiol-containing antioxidants but not non-thiol-containing antioxidants could completely block AF-induced proteasome inhibition, Bcr-Abl downregulation and cell apoptosis (Figure 5B and C). These results provide compelling evidence that proteasome inhibition rather than ROS generation is required for AF-mediated caspase activation and down-regulation of Bcr-Abl in CML cells.

### AF inhibits the growth of Bcr-Abl wild-type and -T315I mutant xenografts in nude mice

**Figure 6:**
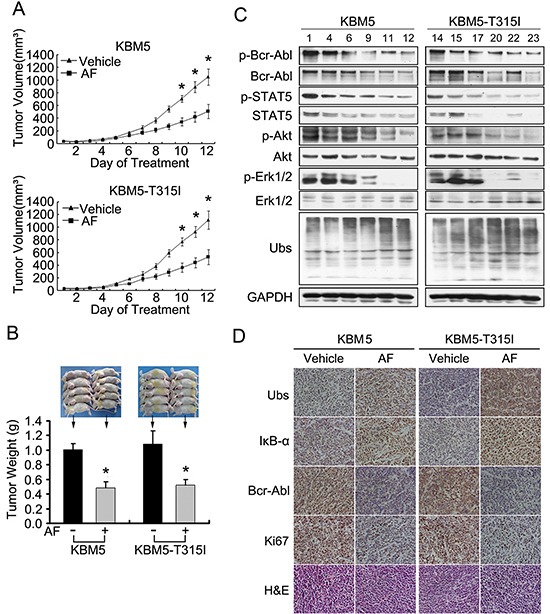
AF inhibits growth of tumor cells in wild type and T315I-mutant Bcr-Abl xenografted mouse model. Nude BALB/c mice bearing KBM5 and KBM5-T315I cells were randomized to vehicle- and AF (7 mg/kg/d)-treatment group Treatment was initiated when the average tumor size reached 50mm^3^. **(A)** Tumor volume was recorded every day after treatment. Mean ±SD. *P < 0.05. *versus* AF treatment. **(B)** Tumor weight on day 12 post initial treatment was calculated and shown. Mean ±SD. *P < 0.05. *versus* vehicle treatment. **(C** and **D)** Western blot and immunohistochemistry analysis were performed to examine ubiquitinated proteins, IκB-α, Bcr-Abl and Ki67 in the tumor tissues. (KBM5 vehicle-treatment group: #1, #4, #6; AF-treatment group: #9, #11, #12; KBM5-T315I vehicle-treatment group: #14, #15, #17; AF-treatment group: #20, #22, #23)

We next evaluate the *in vivo* effect of AF using a nude mouse xenograft model. KBM5 and KMB5-T315I cells were inoculated subcutaneously in nude mice. Mice were then treated with intraperitoneal injections of either vehicle or AF (7 mg/kg/d) for 12 days. It was found that AF treatment significantly inhibited the growth of both Bcr-Abl wild-type and Bcr-Abl-T315I mutant xenografts (Figure 6A); the weights of tumors were significantly reduced in AF-treated group compared to the vehicle-treated (Figure 6B) while body weight remained relatively stable in each group (data not shown). Phospho protein levels of Bcr-Abl and its downstream targets including Akt, Erk1/2 and STAT5 as well as total Bcr-Abl, STAT5 and Akt proteins were significantly decreased in the AF-treated tumors (Figure 6C), while the ubiquitinated proteins and IκB-α were greatly accumulated in AF-treated tumors compared with the control (Figures 6C and D). Immunostaining analysis of Ki67 (a cell proliferation marker) revealed that AF decreased the proliferation of xenografted KBM5 and KBM5-T315I cells (Figure 6D). Together, the results demonstrate that AF can inhibit the *in vivo* tumor growth of xenografted CML cells harboring either wild type Bcr-Abl or the IM-resistant T315I mutant Bcr-Abl.

## DISCUSSION

Acquired resistance to IM mediated by the amplification and mutation of Bcr-Abl presents a great challenge to the therapy of CML [32]. New generations of tyrosine kinase inhibitors have been designed (e.g., nilotinib, dasatinib, sunitinib, ponatinib and GZD824) to overcome the acquired resistance to IM [33, 34]. Although these novel inhibitors can effectively inhibit the phosphorylation of the mutated Bcr-Abl (E255K, M351T), most of them had little effect on Bcr-Abl-T315I. In addition, Bcr-Abl cells are relatively resistant to apoptosis induced by conventional cytotoxic agents [35, 36]. All of these studies find that, CML, being highly dependent on presence of Bcr-Abl, is one of the typical models of oncogene addiction. Bcr-Abl downregulation is the common effect and is likely the major strategy to induce apoptosis in Bcr-Abl-expressing cells.

To search for an alternative therapy for IM-resistant CML, particularly those harboring T315I mutant Bcr-Abl, we evaluated a gold (I) thiolate compound AF, which has been developed for the treatment of rheumatoid arthritis. Recent research found that AF has antitumor activities in a broad range of human cancer cells, including K562 cells expressing wild type Bcr-Abl [37]. In this study, we reported that AF is highly effective in overcoming IM-resistance in cancer cells *in vitro* and *in vivo*. In the *in vitro* study, AF dose- and time-dependently decreased cell viability, induced apoptosis and arrested cell cycle in both IM-sensitive and -resistant cell lines; in the *in vivo* experiment, both IM-sensitive and -resistant xenografted tumors were all sensitive to AF treatment. These results have clearly demonstrated that AF can efficiently induce cytotoxicity in IM-resistant CML cells. To our knowledge, this is the first report to show that AF is effective *in vitro* and *in vivo* against CML cells, including those with the T315I mutation. In present study, we used a solid-tumor model which may be not as good as a non-solid leukemia model. It will be important to test the in vivo effect of AF on a non-solid leukemia model.

Based on these results, KBM5-T315I cells are relatively more sensitive to AF than KBM5 cells. A possible reason for this difference is that KBM5-T315I cells are more malignant than KBM5 cells. It is well known that the ubiquitin-proteasome system is more active in malignant cells than in either less malignant or non-transformed cells [38]; as a result, malignant cells are more sensitive to proteasome inhibition [39]. AF is a proteasomal deubiquitinase inhibitor and is therefore more cytotoxic to KBM5-T315I cells than to KBM5 cells.

**Figure 7:**
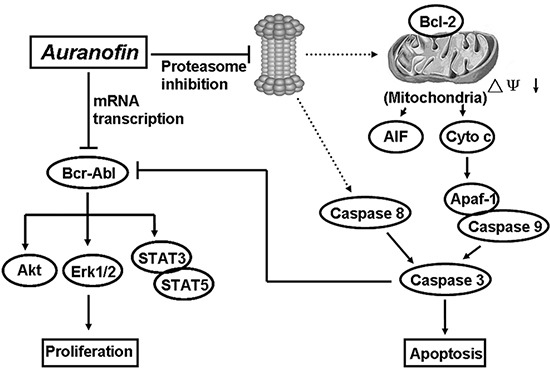
A schematic illustration of a proposed mechanism by which Aurnofin induces cytotoxicity AF induces cytotoxicity *via* both Bcr-Abl mRNA downregulation and proteasome-dependent caspase activation.

Moreover, we have unraveled that AF induces cell apoptosis and overcomes IM-resistance in CML cells through both Bcr/Abl-dependent and -independent mechanisms. On one hand, AF inhibits the gene expression of Bcr-Abl and AF-induced caspase activation cleaves Bcr-Abl (Figure 4A-C), leading to Bcr-Abl downregulation and cell proliferation inhibition; on the other hand, as an inhibitor of proteasome-associated DUBs, AF induced proteasome inhibition-dependent activation of both the intrinsic and the extrinsic caspase pathways (Figure 2 and Figure 5), causing apoptosis (Figure 7). Bcr-Abl is a constitutively active tyrosine kinase that phosphorylates several substrates, and activates multiple signal transduction pathways. The downstream targets of STAT3 and STAT5 responsible for enhanced survival of Bcr-Abl cells are involved in the transcription of Mcl-1, survivin or Bcl-2 [40, 41]. Treatment with AF dose-dependently resulted in down-regulation of anti-apoptotic proteins like Bcl-2, survivin and XIAP, which leads to the decrease of mitochondrial membrane integrity, thus inducing the release of cytochrome C and AIF. The released apoptotic factors either directly or by forming caspase-9 complex, induced caspase activation. These processes have been confirmed in our study. However, we did not see dramatic decreases of Bcr-Abl downstream proteins Mcl-1 and Bcl-xl. The mechanism remains unclear. As these two proteins are also proteasome substrates, therefore we proposed that discrepancy might be due to the balance between protein expression and protein degradation. More studies are needed to delineate the mechanism in the future. AF treatment activated both the extrinsic and intrinsic caspase systems, but caspase-8 activation appears more in parallel to PARP cleavage than caspase-9; therefore, it will be interesting to investigate the direct effect of AF on caspase-8 activation in future studies. The activated caspases, on one hand, induced PARP cleavage and then apoptosis; on the other hand, they cleaved Bcr-Abl protein. This is different from the mechanism mediated by 20S proteasome inhibitors gambogic acid or bortezomib. The 20S proteasome inhibitors have been reported to be effective in overcoming IM-resistant cancer cells *via* different mechanisms. CML cells that express Bcr-Abl are more sensitive to the inhibition of the proteasome with bortezomib than control cells. Bortezomib treatment reduces the proliferation of Bcr-Abl-expressing cells, by inactivating NF-κB and decreasing the phosphorylation of Rb, eventually leading to an increase in caspase-dependent apoptosis [42], which is also correlated with accumulation of cells in the G2/M phase of cell cycle, transient downregulation of NF-κB DNA binding activity, downregulation of Bcl-xL, activation of caspase 3, induction of apoptosis, inhibition of the expression and phosphorylation of Bcr/Abl [27]. Unfortunately, bortezomib had minimal efficacy and considerable toxicity in patients with imatinib-refractory CML [43]. Another proteasome inhibitor gambogic acid was reported to downregulate Bcr-Abl by caspase-dependent Bcr-Abl cleavage and overcome IM-resistance in CML cells [28]. To our knowledge, this is the first report that proteasome-associated DUB inhibition by a clinically used agent can overcome IM-resistance *in vitro* and *in vivo*.

Lastly, we have confirmed in the present study that proteasomal DUB inhibition rather than ROS generation is responsible for AF-induced apoptosis in CML cells. AF itself could increase ROS generation by inhibiting thioredoxin reductase. In most of the previous reports [17, 18, 44], it is believed that ROS elevation is the cause of AF-induced apoptosis, based primarily on that AF induction of apoptosis could be blocked using NAC as the ROS scavenger. Therefore it is reasonable to hypothesize that ROS plays an important role in AF-mediated cell apoptosis in CML cells. However, we and others have reported that thiol-containing antioxidant NAC could both scavenge ROS and directly bind with the active gold atom of AF, thus blocking the active site of AF, including the DUB inhibition activity [26, 45]. To further differentiate the effects of ROS and AF-mediated DUB inhibition, we used two classical antioxidants including thiol-containing (NAC, GEE) and non-thiol-containing (Tbhq, Vitamin C) ones. These four antioxidants could all scavenge AF-induced ROS generation, but based on our results, only thiol-containing antioxidants but not non-thiol-containing antioxidants could prevent AF-induced proteasome inhibition, Bcr-Abl downregulation and apoptosis, demonstrating that DUB inhibition, rather than ROS elevation, is the main mechanism by which AF overcomes IM-resistance in CML cells as we previously reported in other cancer cells [26].

New anticancer drugs are not only time-consuming and costly to develop but also might not outperform existing drugs. To help resolve this dilemma, Blagosklonny has proposed a business model to develop existing drugs for a novel use [46]. The present study has provided strong evidence for this proposition. In summary, we demonstrate here the action and the mechanisms by which AF, an old anti-rheumatic drug, overcomes IM-resistance both in the KBM5-T315I cell cultures and in the KBM5-T315I cell xenografts. Given that AF has an established safety profile in patients, these findings suggest for the first time that AF may have potentially clinical benefit for CML patients, particularly those suffering from imatinib-resistant and recurrent forms of CML, providing great importance in future clinical cancer therapy.

## METHODS

### Materials

AF, N-acetyl-L-cysteine (NAC), Tertiary butylhydroquinone (Tbhq), glutathione ethylene ester (GEE), ascorbic acid (Vitamin C), Annexin V, propidium iodide (PI) and rhodamine-123 were obtained from Sigma-Aldrich (St. Louis, MO). DCF-DA and z-VAD-fmk were from BD Biosciences (San Jose, CA). Antibodies (Abs) against c-Abl (C-19), ubiquitin (P4D1), Mcl-1 (S-19), caspase-3, -8, -9, apoptosis-inducing factor (AIF), Bcl-2, Bax, GAPDH (FL-335) were from Santa Cruz Biotechnology (Santa Cruz, CA). Antibodies against poly (ADP)-ribose polymerase (PARP, clone 4C10-5) was from BD Biosciences. Antibodies against phospho-c-Abl at Y245, phospho-Erk1/2 (T202/Y204), Erk1/2, phospho-Akt, Akt, IκB-α, Bcl-xL, survivin, cleaved caspase-3, -9, cytochrome C, and XIAP were from Cell Signaling Technology (Beverly, MA, USA). Antibodies against phospho-STAT5A/B (Y694/Y699, clone 8-5-2), STAT5, and Ki67 were from Upstate Technology. Enhanced chemiluminescence (ECL) reagents were purchased from Amersham Biosciences (Piscataway, NJ, USA).

### Cell culture

The culture of KBM5 and KBM5-T315I cells was described previously [13, 28, 47]. In brief, KBM5 cells expressing the 210 kDa wild-type Bcr-Abl was derived from a female CML patient. The KBM5-T315I cells, harboring a threonine to isoleucine substitution at position 315 of Abl, were originally established by exposure to increasing concentrations of IM and became IM-resistant. KBM5 cells were cultured in Iscove's modified Dulbecco's medium (Invitrogen) supplemented with 10% heat-inactivated FCS (Hyclone). KBM5-T315I cells were routinely maintained in the same medium but with 1 μmol/L IM, which was removed before experiments with a wash-out period of 2 to 3 days.

### Western blot analysis

Whole cell lysates were prepared in RIPA buffer [13] (1 × PBS, 1% NP-40, 0.5% sodium deoxycholate, 0.1% SDS) supplemented with freshly added 10 mM β-glycerophosphate, 1 mM sodium orthovanadate, 10 mM NaF, 1 mM phenylmethylsulfonyl fluoride (PMSF), and 1 × Roche Complete Mini Protease Inhibitor Cocktail (Roche, Indianapolis, IN). The cytosolic fraction was prepared with digitonin extraction buffer (10 mM PIPES [pH 6.8], 0.015% [wt/vol] digitonin, 300 mM sucrose, 100 mM NaCl, 3 mM MgCl_2_, 5 mM EDTA, and 1 mM phenylmethylsulfonyl fluoride) as described previously to detect the level of cytochrome C and AIF in the cytosol [13]. Western blotting was performed as we previously described [13, 28].

### Cell viability assay

MTS assay (CellTiter 96Aqueous One Solution reagent; Promega, Shanghai, China) was used to test cell viability. 2×10^5^/mL cells in 100 μL were treated with increasing concentrations of AF for 48 hours, control cells received DMSO for a final concentration the same as the highest concentration of AF but less than 0.1%v/v. 4 hours before culture end, 20 μL MTS was added to the wells. The absorbance density was read on a 96-well plate reader at wavelength 490 nm.

### Cell death assay

KBM5 and KBM5-T315I cells were treated with increasing concentrations of AF for indicated time periods, 0.4% Trypan blue was added to detect the number of death cells under the light microscope.

Apoptosis was determined by flow cytometry using Annexin V-fluoroisothiocyanate (FITC)/PI double staining [48, 49]. Cells were incubated with indicated concentration or time duration of AF, collected and washed with binding buffer (BD Biosciences Pharmingen), then incubated in working solution (100 μL bingding buffer with 0.3 μL Annexin V-FITC) for 15 min in dark. Cells were washed and resuspended with binding buffer. PI (Sigma-Aldrich) was added just before flow cytometric analysis.

### Cell cycle analysis

Flow cytometry was used to analyze the cell cycle. After drug treatment, cells were collected and fixed overnight in 66% cold ethanol at −20°C. The cells were then washed twice in cold PBS and stained with propidium iodide for 30 minutes in the dark. Cell cycle distribution was determined using a FACSCalibur flow cytometer with the CellQuest software [13].

### Measurement of mitochondrial membrane integrity

The mitochondrial membrane potential of AF-treated and untreated cells was assayed by using rhodamine-123 (Sigma-Aldrich, St. Louis, MO) staining. Cells were treated with various concentrations of AF for 24 hours and stained with 1 μM of rhodamine-123 for 1 hour at 37°C. Following the staining, the cells were washed and harvested for flow cytometry analysis [28].

### Measurement of reactive oxygen species generation

ROS production was detected as previously reported [26]. Cancer cells were treated with AF (2 μM) in the absence or presence of antioxidants for 1 h. The cells were harvested and incubated with the free serum medium with addition of 10 μM of DCFH-DA for 20 min at 37°C in the dark. In the presence of ROS, DCFH penetrates the cells and is in turn oxidized to DCF. DCF fluorescence was detected by flow cytometry.

### RNA isolation and Real-time quantitative polymerase chain reaction (RT-qPCR)

Total RNA was extracted from 5×10^6^ cells by use of Trizol reagent (Invitrogen). After quantification by spectrophotometry, the first-strand cDNA was synthesized from 500ng of total RNA with the use of the RNA PCR Kit (AMV) Ver.3.0 (TaKaRa, Dalian, China) and random primers. Then 50 ng of total cDNA was use for real-time PCR with the SYBR Premix Ex Taq II Kit (TaKaRa). Each PCR reaction was carried out in a 20 μL volume on 96-well optical reaction plate. The reaction was using the Roche Lighcycler 480 system. The relative gene expression was analyzed by the Comparative Ct method using 18s ribosomal RNA as endogenous control, after confirming that the efficiencies of the target and the endogenous control amplifications were approximately equal. The specific primers for real-time PCR are as follows: Bcr-Abl forward, 5′-AAG CGC AAC AAG CCC ACT GTC TAT-3′; Bcr-Abl reverse, 5′-CTT CGT CTG AGA TAC TGG ATT CCT-3′; 18s forward, 5′-AAA CGG CTA CCA CAT CCA AG-3′; 18s reverse, 5′-CCT CCA ATG GAT CCT CGT TA-3′.

### Nude mouse xenograft model

Nude Balb/c mice were bred at the animal facility of Guangzhou medical university. The mice were housed in barrier facilities with a 12 hours light dark cycle, with food and water available ad libitum. 3×10^7^ of KBM5 or KBM5-T315I cells were inoculated subcutaneously on the flanks of 5-week-old male nude mice as we previously reported [28]. After 72 hours of inoculation, mice were treated with either vehicle (10% DMSO, 30% cremophor and 60% NaCl) or AF (7 mg/kg/day of body weight) for 12 days. Tumors were measured every other day using calipers. Tumor volumes were calculated using the following formula: *a*^2^×*b*×0.4, where *a* is the smallest diameter and *b* is the diameter perpendicular to *a*. The body weight, feeding behavior and motor activity of each animal were monitored as indicators of general health. Tumor xenografts were immediately removed, weighed, fixed, and stored. All animal studies were conducted with the approval of the Guangzhou Medical University Institutional Animal Care and Use Committee.

### Immunohistochemical staining

Immunostaining was performed as we previously reported [28]. Briefly, formalin-fixed xenografts were embedded in paraffin and sectioned according to standard techniques. Tumor xenograft sections (4 μm) were immunostained using the MaxVision kit (MaixinBiol) according to the manufacturer's instructions. The primary antibodies were for ubiquitin, IκB-α, c-Abl, or Ki67. 50 μL MaxVisionTM reagent was applied to each slide. Color was developed with 0.05% diaminobenzidine and 0.03% H_2_O_2_ in 50 mM Tris-HCl (pH 7.6), and the slides were counterstained with hematoxylin. A negative control for every antibody was also included for each xenograft specimen by substituting the primary antibody with preimmune rabbit serum.

### Statistical analysis

All experiments were performed at least thrice, and results are expressed as Mean±SD where applicable. GraphPad Prism 4.0 software (GraphPad Software) was used for statistical analysis. Comparison of multiple groups was made with one-way ANOVA followed by Tukey's test or Newman-Kueuls test. *P* value of <0.05 was considered statistically significant.

## References

[1] Ren R (2005). Mechanisms of BCR-ABL in the pathogenesis of chronic myelogenous leukaemia. Nat Rev Cancer.

[2] Lugo TG, Pendergast AM, Muller AJ, Witte ON (1990). Tyrosine kinase activity and transformation potency of bcr-abl oncogene products. Science.

[3] Gesbert F, Sellers WR, Signoretti S, Loda M, Griffin JD (2000). BCR/ABL regulates expression of the cyclin-dependent kinase inhibitor p27Kip1 through the phosphatidylinositol 3-Kinase/AKT pathway. J Biol Chem.

[4] Danial NN, Rothman P (2000). JAK-STAT signaling activated by Abl oncogenes. Oncogene.

[5] Amarante-Mendes GP, McGahon AJ, Nishioka WK, Afar DE, Witte ON, Green DR (1998). Bcl-2-independent Bcr-Abl-mediated resistance to apoptosis: protection is correlated with up regulation of Bcl-xL. Oncogene.

[6] Soliera AR, Mariani SA, Audia A, Lidonnici MR, Addya S, Ferrari-Amorotti G, Cattelani S, Manzotti G, Fragliasso V, Peterson L, Perini G, Holyoake TL, Calabretta B (2012). Gfi-1 inhibits proliferation and colony formation of p210BCR/ABL-expressing cells via transcriptional repression of STAT 5 and Mcl-1. Leukemia.

[7] Airiau K, Mahon FX, Josselin M, Jeanneteau M, Turcq B, Belloc F (2012). ABT-737 increases tyrosine kinase inhibitor-induced apoptosis in chronic myeloid leukemia cells through XIAP downregulation and sensitizes CD34(+) CD38(−) population to imatinib. Exp Hematol.

[8] Thienelt CD, Green K, Bowles DW (2012). New and established tyrosine kinase inhibitors for chronic myeloid leukemia. Drugs Today (Barc).

[9] Gora-Tybor J, Robak T (2008). Targeted drugs in chronic myeloid leukemia. Curr Med Chem.

[10] Alvarado Y, Kantarjian H, O'Brien S, Faderl S, Borthakur G, Burger J, Wierda W, Garcia-Manero G, Shan J, Cortes J (2009). Significance of suboptimal response to imatinib, as defined by the European LeukemiaNet, in the long-term outcome of patients with early chronic myeloid leukemia in chronic phase. Cancer.

[11] Kantarjian HM, Talpaz M, Giles F, O'Brien S, Cortes J (2006). New insights into the pathophysiology of chronic myeloid leukemia and imatinib resistance. Ann Intern Med.

[12] Shah NP (2005). Loss of response to imatinib: mechanisms and management. Hematology Am Soc Hematol Educ Program.

[13] Shi X, Jin Y, Cheng C, Zhang H, Zou W, Zheng Q, Lu Z, Chen Q, Lai Y, Pan J (2009). Triptolide inhibits Bcr-Abl transcription and induces apoptosis in STI571-resistant chronic myelogenous leukemia cells harboring T315I mutation. Clin Cancer Res.

[14] Kaur P, Feldhahn N, Zhang B, Trageser D, Muschen M, Pertz V, Groffen J, Heisterkamp N (2007). Nilotinib treatment in mouse models of P190 Bcr/Abl lymphoblastic leukemia. Mol Cancer.

[15] Talpaz M, Shah NP, Kantarjian H, Donato N, Nicoll J, Paquette R, Cortes J, O'Brien S, Nicaise C, Bleickardt E, Blackwood-Chirchir MA, Iyer V, Chen TT, Huang F, Decillis AP, Sawyers CL (2006). Dasatinib in imatinib-resistant Philadelphia chromosome-positive leukemias. N Engl J Med.

[16] Morinaga K, Yamauchi T, Kimura S, Maekawa T, Ueda T (2008). Overcoming imatinib resistance using Src inhibitor CGP76030, Abl inhibitor nilotinib and Abl/Lyn inhibitor INNO-406 in newly established K562 variants with BCR-ABL gene amplification. Int J Cancer.

[17] Cortes JE, Kim DW, Pinilla-Ibarz J, le Coutre P, Paquette R, Chuah C, Nicolini FE, Apperley JF, Khoury HJ, Talpaz M, DiPersio J, DeAngelo DJ, Abruzzese E, Rea D, Baccarani M, Muller MC, Gambacorti-Passerini C, Wong S, Lustgarten S, Rivera VM, Clackson T, Turner CD, Haluska FG, Guilhot F, Deininger MW, Hochhaus A, Hughes T, Goldman JM, Shah NP, Kantarjian H (2013). A phase 2 trial of ponatinib in Philadelphia chromosome-positive leukemias. N Engl J Med.

[18] Shaw IC (1999). Gold-based therapeutic agents. Chem Rev.

[19] Ashino T, Sugiuchi J, Uehara J, Naito-Yamamoto Y, Kenmotsu S, Iwakura Y, Shioda S, Numazawa S, Yoshida T (2011). Auranofin protects against cocaine-induced hepatic injury through induction of heme oxygenase-1. J Toxicol Sci.

[20] Fiskus W, Saba N, Shen M, Ghias M, Liu J, Gupta SD, Chauhan L, Rao R, Gunewardena S, Schorno K, Austin CP, Maddocks K, Byrd J, Melnick A, Huang P, Wiestner A, Bhalla KN (2014). Auranofin induces lethal oxidative and endoplasmic reticulum stress and exerts potent preclinical activity against chronic lymphocytic leukemia. Cancer Res.

[21] Kim IS, Jin JY, Lee IH, Park SJ (2004). Auranofin induces apoptosis and when combined with retinoic acid enhances differentiation of acute promyelocytic leukaemia cells in vitro. Br J Pharmacol.

[22] Berners-Price SJ, Filipovska A (2011). Gold compounds as therapeutic agents for human diseases. Metallomics.

[23] Marzano C, Gandin V, Folda A, Scutari G, Bindoli A, Rigobello MP (2007). Inhibition of thioredoxin reductase by auranofin induces apoptosis in cisplatin-resistant human ovarian cancer cells. Free Radic Biol Med.

[24] Glennas A, Kvien TK, Andrup O, Clarke-Jenssen O, Karstensen B, Brodin U (1997). Auranofin is safe and superior to placebo in elderly-onset rheumatoid arthritis. Br J Rheumatol.

[25] Debnath A, Parsonage D, Andrade RM, He C, Cobo ER, Hirata K, Chen S, Garcia-Rivera G, Orozco E, Martinez MB, Gunatilleke SS, Barrios AM, Arkin MR, Poole LB, McKerrow JH, Reed SL (2012). A high-throughput drug screen for Entamoeba histolytica identifies a new lead and target. Nat Med.

[26] Liu N, Li X, Huang H, Zhao C, Liao S, Yang C, Liu S, Song W, Lu X, Lan X, Chen X, Yi S, Li X, Jiang L, Zhao C, Dong X, Zhou P, Li S, Wang S, Shi X, Dou QP, Wang X, Liu J (2014). Clinically used antirheumatic agent auranofin is a proteasomal deubiquitinase inhibitor and inhibits tumor growth. Oncotarget.

[27] Gatto S, Scappini B, Pham L, Onida F, Milella M, Ball G, Ricci C, Divoky V, Verstovsek S, Kantarjian HM, Keating MJ, Cortes-Franco JE, Beran M (2003). The proteasome inhibitor PS-341 inhibits growth and induces apoptosis in Bcr/Abl-positive cell lines sensitive and resistant to imatinib mesylate. Haematologica.

[28] Shi X, Chen X, Li X, Lan X, Zhao C, Liu S, Huang H, Liu N, Liao S, Song W, Zhou P, Wang S, Xu L, Wang X, Dou QP, Liu J (2014). Gambogic acid induces apoptosis in imatinib-resistant chronic myeloid leukemia cells via inducing proteasome inhibition and caspase-dependent Bcr-Abl downregulation. Clin Cancer Res.

[29] Di Bacco AM, Cotter TG (2002). p53 expression in K562 cells is associated with caspase-mediated cleavage of c-ABL and BCR-ABL protein kinases. Br J Haematol.

[30] Omata Y, Lewis JB, Lockwood PE, Tseng WY, Messer RL, Bouillaguet S, Wataha JC (2006). Gold-induced reactive oxygen species (ROS) do not mediate suppression of monocytic mitochondrial or secretory function. Toxicol In Vitro.

[31] Watson WH, Heilman JM, Hughes LL, Spielberger JC (2008). Thioredoxin reductase-1 knock down does not result in thioredoxin-1 oxidation. Biochem Biophys Res Commun.

[32] McGahon A, Bissonnette R, Schmitt M, Cotter KM, Green DR, Cotter TG (1994). BCR-ABL maintains resistance of chronic myelogenous leukemia cells to apoptotic cell death. Blood.

[33] Beyazit Y, Kekilli M, Haznedaroglu IC (2010). Second-generation BCR-ABL kinase inhibitors in CML. N Engl J Med.

[34] Ren X, Pan X, Zhang Z, Wang D, Lu X, Li Y, Wen D, Long H, Luo J, Feng Y, Zhuang X, Zhang F, Liu J, Leng F, Lang X, Bai Y, She M, Tu Z, Pan J, Ding K (2013). Identification of GZD824 as an orally bioavailable inhibitor that targets phosphorylated and nonphosphorylated breakpoint cluster region-Abelson (Bcr-Abl) kinase and overcomes clinically acquired mutation-induced resistance against imatinib. J Med Chem.

[35] Bedi A, Barber JP, Bedi GC, el-Deiry WS, Sidransky D, Vala MS, Akhtar AJ, Hilton J, Jones RJ (1995). BCR-ABL-mediated inhibition of apoptosis with delay of G2/M transition after DNA damage: a mechanism of resistance to multiple anticancer agents. Blood.

[36] Bueno-da-Silva AE, Brumatti G, Russo FO, Green DR, Amarante-Mendes GP (2003). Bcr-Abl-mediated resistance to apoptosis is independent of constant tyrosine-kinase activity. Cell Death Differ.

[37] Liu JJ, Liu Q, Wei HL, Yi J, Zhao HS, Gao LP (2011). Inhibition of thioredoxin reductase by auranofin induces apoptosis in adriamycin-resistant human K562 chronic myeloid leukemia cells. Pharmazie.

[38] Daniel KG, Gupta P, Harbach RH, Guida WC, Dou QP (2004). Organic copper complexes as a new class of proteasome inhibitors and apoptosis inducers in human cancer cells. Biochem Pharmacol.

[39] Adams J (2004). The development of proteasome inhibitors as anticancer drugs. Cancer cell.

[40] Warsch W, Kollmann K, Eckelhart E, Fajmann S, Cerny-Reiterer S, Holbl A, Gleixner KV, Dworzak M, Mayerhofer M, Hoermann G, Herrmann H, Sillaber C, Egger G, Valent P, Moriggl R, Sexl V (2011). High STAT5 levels mediate imatinib resistance and indicate disease progression in chronic myeloid leukemia. Blood.

[41] Nelson EA, Walker SR, Weisberg E, Bar-Natan M, Barrett R, Gashin LB, Terrell S, Klitgaard JL, Santo L, Addorio MR, Ebert BL, Griffin JD, Frank DA (2011). The STAT5 inhibitor pimozide decreases survival of chronic myelogenous leukemia cells resistant to kinase inhibitors. Blood.

[42] Albero MP, Vaquer JM, Andreu EJ, Villanueva JJ, Franch L, Ivorra C, Poch E, Agirre X, Prosper F, Perez-Roger I (2010). Bortezomib decreases Rb phosphorylation and induces caspase-dependent apoptosis in Imatinib-sensitive and -resistant Bcr-Abl1-expressing cells. Oncogene.

[43] Santos FP, Kantarjian H, McConkey D, O'Brien S, Faderl S, Borthakur G, Ferrajoli A, Wright J, Cortes J (2011). Pilot study of bortezomib for patients with imatinib-refractory chronic myeloid leukemia in chronic or accelerated phase. Clin Lymphoma Myeloma Leuk.

[44] Kim NH, Park HJ, Oh MK, Kim IS (2013). Antiproliferative effect of gold(I) compound auranofin through inhibition of STAT3 and telomerase activity in MDA-MB 231 human breast cancer cells. BMB Rep.

[45] Albert A, Brauckmann C, Blaske F, Sperling M, Engelhard C, Karst U (2012). Speciation analysis of the antirheumatic agent Auranofin and its thiol adducts by LC/ESI-MS and LC/ICP-MS. J Anal At Spectrom.

[46] Blagosklonny MV (2003). A new science-business paradigm in anticancer drug development. Trends Biotechnol.

[47] Xu F, Shi X, Li S, Cui J, Lu Z, Jin Y, Lin Y, Pang J, Pan J (2010). Design, synthesis, and biological evaluation of novel water-soluble triptolide derivatives: Antineoplastic activity against imatinib-resistant CML cells bearing T315I mutant Bcr-Abl. Bioorg Med Chem.

[48] Huang H, Zhang X, Li S, Liu N, Lian W, McDowell E, Zhou P, Zhao C, Guo H, Zhang C, Yang C, Wen G, Dong X, Lu L, Ma N, Dong W, Dou QP, Wang X, Liu J (2010). Physiological levels of ATP negatively regulate proteasome function. Cell Res.

[49] Huang H, Liu N, Guo H, Liao S, Li X, Yang C, Liu S, Song W, Liu C, Guan L, Li B, Xu L, Zhang C, Wang X, Dou QP, Liu J (2012). L-carnitine is an endogenous HDAC inhibitor selectively inhibiting cancer cell growth in vivo and in vitro. PLoS One.

